# The Cardiovascular and Cerebrovascular Health in North China From 2006 to 2011: Results From the KaiLuan Study

**DOI:** 10.3389/fcvm.2021.683416

**Published:** 2021-07-12

**Authors:** Yao Yu, Zhiyi Dong, Yongjie Li, Jun Zhang, Sufeng Yin, Xuguang Gao, Shouling Wu, KaiLuan Study Investigators

**Affiliations:** ^1^Department of Neurology, Peking University People's Hospital, Beijing, China; ^2^Department of Biomedical Engineering, Stony Brook University, Stony Brook, NY, United States; ^3^Department of Preventive Medicine, School of Public Health, North China University of Science and Technology, Tangshan, China; ^4^Department of Cardiology, Kailuan General Hospital, North China University of Science and Technology, Tangshan, China

**Keywords:** epidemiology, cardiovascular and cerebrovascular disease risk factor, prevention, trends, Kailuan study

## Abstract

**Background:** The American Heart Association (AHA) defined cardiovascular health in terms of four behaviors (smoking, diet, physical activity, body weight) and three factors (plasma glucose, cholesterol, blood pressure). By this definition, the prevalence of ideal cardiovascular health behaviors and factors is negatively correlated with all-cause mortality and risks of cardiovascular and cerebrovascular diseases and malignancy. We analyzed the trends in cardiovascular and cerebrovascular health behaviors and factors in the population of the KaiLuan study for 2006–2011, reported the results, and provided evidence for prevention.

**Methods and Results:** We calculated the prevalence of cardiovascular and cerebrovascular health behaviors and factors from KaiLuan data for 2006–2007, 2008–2009, and 2010–2011. The prevalence of ideal cardiovascular and cerebrovascular health behaviors and factors is low in the KaiLuan population.

**Conclusions:** The prevalence of ideal cardiovascular and cerebrovascular health behaviors and factors is low in the KaiLuan population.

**Clinical Trial Registration:** http://www.chictr.org/cn/proj/show.aspx?proj=1441, unique identifier: ChiCTR-TNC-11001489.

## Introduction

The American Heart Association (AHA) defined four cardiovascular health behaviors (smoking, diet, physical activity, body weight) and three health factors (plasma glucose, cholesterol, blood pressure) based on epidemiologic data (1). By this definition, the prevalence of ideal cardiovascular health behaviors and factors is negatively correlated with all-cause mortality and risks of cardiovascular disease and malignancy (1–10). Recently, because of lifestyle improvements, the incidence of cardiovascular and cerebrovascular diseases has shown a downward trend (11, 12).

Ideal cardiovascular and cerebrovascular health metrics are also protective against cerebrovascular disease (13–15). Zeng et al. reported their prevalence in the Chinese population (16, 17); such findings suggest that the prevalence of ideal AHA behaviors and factors is low among the Chinese population, and we speculate that this is one reason why the incidence of mortality from chronic non-communicable diseases has increased in China recently (18, 19).

The incidence of cardiovascular and cerebrovascular events in the Chinese population in the future will be determined by cardiovascular health behaviors and factors. We analyzed the trends among the population of the KaiLuan study for 2006–2011, reported the results, and provided evidence for the future.

## Methods

### Subjects

The study was approved by the KaiLuan General Hospital Ethics Committee. The KaiLuan study began in July 2006 with a prospective cohort based on the KaiLuan community focusing on risk factors and interventions for cardiologic, cerebrovascular, and related diseases. The KaiLuan Group Co., Ltd. produces coal and chemical industrial products; it is located in the city of Tangshan in the center of the Bohai Sea Gulf region and has 150,000 serving and retired employees. The KaiLuan community is owned and managed by the KaiLuan Group and has 11 hospitals responsible for its healthcare (13). Employees undergo health examination funded by the KaiLuan Group every 2 years; data are now available for 2006–2007, 2008–2009, and 2010–2011.

The inclusion criteria for this study were as follows: age ≥18 years, cognitive ability sufficient to complete the questionnaire oneself, and provision of informed consent. The exclusion criteria were as follows: history of stroke or myocardial infarction, glomerular filtration rate <30 ml/min/1.73 m^2^, or data related to cardiovascular health behaviors and factors missing.

### Definition of Cardiovascular and Cerebrovascular Health Metrics

The cardiovascular and cerebrovascular health behaviors and factors investigated in this study are those defined by the AHA (1). Because the questionnaire used in the KaiLuan study did not ask about vegetable intake and the China National Nutrition and Health Survey shows that only 18.4% of Chinese people had daily salt intake <6 g in 2002, salt preference was used as a surrogate for the diet metric of AHA. Because the amount of salt consumed was not measured precisely, salt preference was classified as “low,” “medium,” or “high,” with low substituted for the ideal level of the metric of AHA (15). Cardiovascular and cerebrovascular health metrics as defined by the 2020 Strategic Impact Goals Committee of AHA and the KaiLuan study are displayed in Tables 1, 2. The histories of hypertension, diabetes mellitus, stroke, and myocardial infarction of the subjects were determined from the survey questionnaire.

**Table 1:** Definitions of cardiovascular health metrics from the 2020 Strategic Impact Goals Committee of the American Heart Association.

		**Level of cardiovascular health**	
**Metric**	**Poor**	**Intermediate**	**Ideal**
Smoking	Current smoker	Quit smoking <12 months ago	Never smoker or quit smoking ≥12 months ago
Diet^a^	Diet score = 0/1	Diet score = 2/3	Diet score = 4/5
Physical activity^b^	No physical activity	1–149 min/week moderate intensity or 1–74 min/week vigorous intensity or 1–149 min/week moderate and vigorous	≥150 min/week moderate intensity or ≥75 min/week vigorous intensity or ≥150 min/week moderate and vigorous
Body weight	BMI ≥30 kg/m^2^	BMI 25–29.9 kg/m^2^	BMI <25 kg/m^2^
Fasting plasma glucose	≥126 mg/dl	100–125 mg/dl or treated to goal	<100 mg/dl
Total cholesterol	≥240 mg/dl	200–239 or treated to goal	<200 mg/dl
Blood pressure	SBP ≥140 mm Hg or DBP ≥90 mm Hg	SBP 120–139 mm Hg or DBP 80–89 mm Hg or treated to goal	<120/ <80 mm Hg

*BMI, body mass index; SBP, systolic blood pressure; DBP, diastolic blood pressure*.

a *Diet score (scale: 0–5) calculated based on one point for each of the five components: ≥4.5 cups per day fruits/vegetables; ≥two 3.5 oz. servings of fish per week; <1,500 mg/day sodium; ≤ 450 kcal (36 oz.) per week sweets/sugar-sweetened beverages; and ≥three servings per day whole grains (1.1 g of fiber in 10 g of carbohydrate; 1 oz. equivalent servings)*.

b *Leisure time physical activity*.

**Table 2:** Definitions of cardiovascular and cerebrovascular health metrics from the KaiLuan study.

		**Level of cardiovascular health**	
**Metric**	**Poor**	**Intermediate**	**Ideal**
Smoking	Current smoker	Quit smoking <12 months ago	Never smoker or quit smoking ≥12 months ago
Salt preference	High	Medium	Low
Physical activity	Inactive (none)	Moderately active	Very active (≥three times/week and ≥30 min each time)
Body weight	BMI ≥ 30 kg/m^2^	BMI 25–29.9 kg/m^2^	BMI <25 kg/m^2^
Fasting plasma glucose	≥126 mg/dl	100–125 mg/dl or treated to goal	<100 mg/dl
Total cholesterol	≥240 mg/dl	200–239 mg/dl or treated to goal	<200 mg/dl
Blood pressure	SBP ≥ 140 mm Hg or DBP ≥ 90 mm Hg	SBP 120–139 mm Hg or DBP 80–89 mm Hg or treated to goal	<120/ <80 mm Hg

*BMI, body mass index; SBP, systolic blood pressure; DBP, diastolic blood pressure*.

### Survey Questionnaire and Anthropometric Measurements

Details of these procedures have been published previously (13, 20–22).

### Statistics

Statistical analyses were performed using SPSS for Windows v13.0 (SPSS Inc., Chicago, IL, USA). Continuous variables were described by the mean ± standard deviation and categorical variables by percentages.

## Results

The numbers of employees who participated in the 2006–2007, 2008–2009, and 2010–2011 health examinations were 101,510, 101,133, and 92,967, respectively. The following numbers were excluded from our study: 10,126 from 2006 to 2007 (2,556 history of stroke, 1,316 history of myocardial infarction, 563 glomerular filtration rate <30 ml/min/1.73 m^2^, 6,051 incomplete data), 14,657 from 2008 to 2009 (2,417 history of stroke, 1,033 history of myocardial infarction, 287 glomerular filtration rate <30 ml/min/1.73 m^2^, 11,506 incomplete data), and 11,050 from 2010 to 2011 (2,362 history of stroke, 1,308 history of myocardial infarction, 170 glomerular filtration rate <30 ml/min/1.73 m^2^, 7,681 incomplete data). Ultimately, 91,384, 86,476, and 81,917 subjects, respectively, were included in the study; 79.39% (72,552), 78.92% (68,245), and 78.54% (64,339) were male. Their average ages were 50.47 ± 12.35, 50.71 ± 12.48, and 51.32 ± 12.95 years.

The prevalence of cardiovascular and cerebrovascular health metrics categorized as poor, intermediate, or ideal for 2006–2007, 2008–2009, and 2010–2011 is displayed by sex in Tables 3, 4.

**Table 3:** Distribution (2006–2007, 2008–2009, and 2010–2011) of poor, intermediate, and ideal levels of cardiovascular health metrics for men (KaiLuan study).

	**2006–2007**	**2008–2009**	**2010–2011**
**Smoking**			
Poor	44.76	52.37	45.03
Intermediate	5.80	5.5	5.26
Ideal	49.44	42.13	49.71
**Salt**			
Poor	11.75	12.95	11.93
Intermediate	79.18	73.59	70.36
Ideal	9.07	13.46	17.71
**Physical activity**			
Poor	11.01	22.45	33.37
Intermediate	76.04	61.61	54.17
Ideal	12.94	15.94	12.47
**Body weight**			
Poor	8.47	7.83	8.31
Intermediate	41.60	40.22	41.64
Ideal	49.93	51.95	50.04
**Glucose**			
Poor	6.99	7.7	6.90
Intermediate	22.86	25.99	26.79
Ideal	70.15	66.31	66.31
**Total cholesterol**			
Poor	9.65	9.68	7.69
Intermediate	26.56	25.6	23.00
Ideal	63.79	64.72	69.31
**Blood pressure**			
Poor	38.83	40.89	37.17
Intermediate	41.01	42.45	46.80
Ideal	20.16	16.66	16.03

**Table 4:** Distribution (2006–2007, 2008–2009, and 2010–2011) of poor, intermediate, and ideal levels of cardiovascular and cerebrovascular health metrics for women (KaiLuan study).

	**2006–2007**	**2008–2009**	**2010–2011**
**Smoking**			
Poor	1.81	1.54	1.09
Intermediate	0.39	0.26	0.22
Ideal	97.8	98.2	98.69
**Salt**			
Poor	6.67	8.35	6.88
Intermediate	83.96	75.09	75.37
Ideal	9.38	16.55	17.76
**Physical activity**			
Poor	4.80	17.6	30.83
Intermediate	83.65	66.94	58.05
Ideal	11.55	15.46	11.12
**Body weight**			
Poor	7.63	6.36	6.56
Intermediate	30.49	29.05	29.54
Ideal	61.88	64.59	63.90
**Glucose**			
Poor	5.86	5.62	5.19
Intermediate	15.76	17.86	19.47
Ideal	78.38	76.52	75.34
**Total cholesterol**			
Poor	9.44	8.48	8.42
Intermediate	26.11	23.29	22.25
Ideal	64.46	68.23	69.34
**Blood pressure**			
Poor	24.99	22.33	21.75
Intermediate	37.04	35.13	40.17
Ideal	37.96	42.55	38.09

In this study, 57,659 individuals participated in all three health examinations. We analyzed the data for these individuals only using the methods above and obtained similar results (Supplementary Tables 1, 2).

## Discussion

The cardiovascular and cerebrovascular health behaviors and factors in the KaiLuan population show two characteristics for 2006–2011. First, the prevalence of the ideal level of each metric is low; for salt preference and physical activity, it is <20%. In the USA, only the prevalence of ideal diet is <1%; the ideal levels of the other six metrics are >35%. Second, the prevalence of the poor level of each metric is high: up to 30% for poor blood pressure and around 10% for the other six. In the USA, with the exception of diet and physical activity, the poor level prevalence is about 10%. If we had defined these two metrics according to the AHA, the prevalence of their ideal levels would be much lower and those of their poor levels much higher.

A high salt diet is a risk factor for cardiovascular diseases such as hypertension (23). In 2007, the World Health Organization (WHO) recommended a daily salt intake of <5 g (24). The China National Nutrition and Health Survey showed that only 18% of the population consumed <6 g of salt daily. The prevalence of ideal salt preference is about 17% in 2011. We must improve this situation and it is important for the prevention and control of cardiovascular and cerebrovascular diseases. Salt-sensitive hypertension accounts for 60% of the cases of hypertension in China (25), and a study has indicated that reducing salt consumption by 15%, accompanied by tobacco control, will save 45 million lives at a cost of <3 yuan per person (US$0.4) (26).

Smoking is related to many conditions and is a risk factor for cardiovascular disease (27, 28), the incidence of which declines with quitting smoking (29–31). For patients with cardiovascular disease, quitting smoking leads to a greater all-cause mortality reduction than controlling blood pressure, total cholesterol, and other risk factors, though its effect is less than that of aspirin (32). China is a major consumer of tobacco (33). The number of male smokers is up to 300 million, accounting for one-third of the total number worldwide. The annual economic burden due to smoking is ~US$350 million (34). For men, the prevalence of poor smoking level was up to 45% in 2011. Further strict controls on tobacco should be established to achieve health goals (35).

In 2011, the prevalence of physical inactivity is about 30%. Physical activity is an important cardiovascular health behavior, improving not only blood pressure but also fasting plasma glucose, total cholesterol, and other health indicators (36, 37). There is a significant relationship between frequency of exercise and medical costs incurred (38). Weight gain and increased total cholesterol and fasting plasma glucose resulting from physical inactivity will weaken or even counteract the benefits from the improvement of other cardiovascular health metrics. In this era of readily available transportation, mechanization in the workplace, and television and the internet at home, how to improve the willingness of citizens to participate in physical exercise is a challenge that must be addressed.

In 2011, the prevalence of the ideal levels of total cholesterol and BMI is more than 50%. The prevalence of poor levels of fasting plasma glucose is about 7%. It will be necessary to increase publicity and improve education at both the individual and population levels to increase physical activity and halt this trend.

There is a linear relationship between blood pressure and the incidence of cardiovascular and cerebrovascular diseases, with the risk of these conditions increasing with rising blood pressure above 115/75 mm Hg (39, 40). Blood pressure is one of the most important risk factors for cardiovascular disease and its control is crucial (41–43). Early intervention for hypertension and its complications can reduce the high cost of treatment and improve quality of life caused by cardiovascular and cerebrovascular diseases and other chronic non-communicable conditions (44, 45). In 2011, the prevalence of poor levels of blood pressure is up to 40% in men. Measures should be taken to control the situation.

In 2013, the WHO issued a draft action plan for the prevention and control of non-communicable diseases in 2013–2020 and a draft comprehensive global monitoring framework and targets. It was determined that agencies of the WHO and the United Nations and other international partners will take action together to achieve goals of reducing the prevalence of smoking by 15% among people aged ≥15 years, the average intake of salt/sodium by 30%, and the prevalence of physical inactivity by 10%; controlling the increase of diabetes and obesity; controlling or reducing the prevalence of high blood pressure by 25%; and reducing the risk of early death from cardiovascular disease and other chronic non-communicable diseases by 25% by 2025 (46, 47). The Chinese government proposed a Health China 2020 strategy (35) with the goals of controlling the prevalence of smoking at 40% for men and 4% for women (aged ≥15 years), reducing daily sodium intake to 8 g, maintaining the prevalence of frequent participation in physical activity at ≥40% of the population (aged ≥8 years), controlling the prevalence of overweight adults at 25%, controlling the prevalence of high fasting plasma glucose with diabetes at 45%, controlling the prevalence of hypertension at 30%, and reducing mortality from cardiovascular disease by 30%.

It should be noted that we have undertaken health education in 11,679 patients with hypertension among the study population who met certain criteria, measuring their blood pressure every 2 weeks and administering nitrendipine, hydrochlorothiazide, captopril, and spironolactone antihypertensive drugs without charge since April 2009. These interventions may have influenced the results of the present study; the real situation may thus be worse.

## Data Availability Statement

The raw data supporting the conclusions of this article will be made available by the authors, without undue reservation.

## Author Contributions

All authors listed have made a substantial, direct and intellectual contribution to the work, and approved it for publication.

## Conflict of Interest

The authors declare that the research was conducted in the absence of any commercial or financial relationships that could be construed as a potential conflict of interest.
