# Supplementary material for: The Cardiovascular and Cerebrovascular Health in North China From 2006 to 2011: Results From the KaiLuan Study
Source: Front Cardiovasc Med. 2021 Jul 12;8:683416. doi: 10.3389/fcvm.2021.683416 (PMC8310945; doi:10.3389/fcvm.2021.683416)
Supplement: Supplementary file 1 [file Table_1.docx]

**Supplemental Table 1. Distribution (2006–2007 and 2010–2011) of Poor, Intermediate and Ideal Levels of Cardiovascular and Cerebrovascular Health Metrics for Men: 57,659 Subjects from the KaiLuan Study**

|  | 2006–2007 | 2010–2011 |
| --- | --- | --- |
| Smoking |  |  |
| Poor | 44.62 | 46.39 |
| Intermediate | 6.11 | 5.44 |
| Ideal | 49.27 | 48.17 |
| Salt |  |  |
| Poor | 11.54 | 11.83 |
| Intermediate | 79.12 | 70.52 |
| Ideal | 9.34 | 17.65 |
| Physical activity |  |  |
| Poor | 11.13 | 33.87 |
| Intermediate | 75.75 | 54 |
| Ideal | 13.12 | 12.22 |
| Body weight |  |  |
| Poor | 8.32 | 8.6 |
| Intermediate | 42.5 | 42.74 |
| Ideal | 49.17 | 49.4 |
| Glucose |  |  |
| Poor | 6.7 | 7.15 |
| Intermediate | 23.31 | 28.19 |
| Ideal | 69.99 | 64.67 |
| Total cholesterol |  |  |
| Poor | 9.48 | 8.31 |
| Intermediate | 26.32 | 23.63 |
| Ideal | 64.2 | 68.33 |
| Blood pressure |  |  |
| Poor | 37.35 | 38.61 |
| Intermediate | 42.11 | 46.98 |
| Ideal | 20.55 | 14.68 |
