# Supplementary material for: The Cardiovascular and Cerebrovascular Health in North China From 2006 to 2011: Results From the KaiLuan Study
Source: Front Cardiovasc Med. 2021 Jul 12;8:683416. doi: 10.3389/fcvm.2021.683416 (PMC8310945; doi:10.3389/fcvm.2021.683416)
Supplement: Supplementary file 2 [file Table_2.docx]

**Supplemental Table 2. Distribution (2006–2007 and 2010–2011) of Poor, Intermediate and Ideal Levels of Cardiovascular and Cerebrovascular Health Components for Women: 57,659 Subjects from the KaiLuan Study**

|  | 2006–2007 | 2010–2011 |
| --- | --- | --- |
| Smoking |  |  |
| Poor | 1.4 | 1.18 |
| Intermediate | 0.41 | 0 |
| Ideal | 98.19 | 98.82 |
| Salt |  |  |
| Poor | 6.54 | 7.26 |
| Intermediate | 84.33 | 75.47 |
| Ideal | 9.13 | 18.09 |
| Physical activity |  |  |
| Poor | 4.64 | 30.61 |
| Intermediate | 83.36 | 58.13 |
| Ideal | 12 | 11.83 |
| Body weight |  |  |
| Poor | 7.58 | 6.51 |
| Intermediate | 30.82 | 30.76 |
| Ideal | 61.6 | 62.73 |
| Glucose |  |  |
| Poor | 5.56 | 5.22 |
| Intermediate | 15.54 | 20 |
| Ideal | 78.9 | 74.52 |
| Total cholesterol |  |  |
| Poor | 9.25 | 9.3 |
| Intermediate | 26.47 | 22.85 |
| Ideal | 64.28 | 67.85 |
| Blood pressure |  |  |
| Poor | 24.55 | 21.72 |
| Intermediate | 38.53 | 40.49 |
| Ideal | 36.92 | 37.78 |
